# Application of the SSB biosensor to study *in vitro* transcription

**DOI:** 10.1016/j.bbrc.2018.01.147

**Published:** 2018-02-12

**Authors:** Alexander Cook, Yukti Hari-Gupta, Christopher P. Toseland

**Affiliations:** School of Biosciences, University of Kent, Canterbury, CT2 7NJ, UK

**Keywords:** Biosensor, Transcription, SSB, Myosin, RNA polymerase, Gene expression, mRNA, SSB, single stranded binding protein, IDCC, *N*-[2-(iodoacetamido)ethyl]-7-diethylaminocoumarin-3-carboxamide), MDCC, *N*-[2-(1-maleimidyl)ethyl]-7-diethylaminocoumarin-3-carboxamide, RNAP, RNA Polymerase

## Abstract

Gene expression, catalysed by RNA polymerases (RNAP), is one of the most fundamental processes in living cells. The majority of methods to quantify mRNA are based upon purification of the nucleic acid which leads to experimental inaccuracies and loss of product, or use of high cost dyes and sensitive spectrophotometers. Here, we describe the use of a fluorescent biosensor based upon the single stranded binding (SSB) protein. In this study, the SSB biosensor showed similar binding properties to mRNA, to that of its native substrate, single-stranded DNA (ssDNA). We found the biosensor to be reproducible with no associated loss of product through purification, or the requirement for expensive dyes. Therefore, we propose that the SSB biosensor is a useful tool for comparative measurement of mRNA yield following *in vitro* transcription.

## Highlights

• Single-stranded binding protein can bind mRNA similar to single-stranded DNA.
• The biosensor MDCC-SSB can be used to quantify mRNA yield from *in vitro* transcription.
• Myosin VI motor activity is required for *in vitro* and *in vivo* transcription.

## 1 Introduction

The information to develop and sustain life is encoded within DNA. RNAPs facilitate the distribution of this information through the transcription of DNA into mRNA. *In vitro* transcription assays have allowed the biochemical characterisation of these complex multi-protein machines. Classically, in order to measure the transcriptional activity of RNAPII, *in vitro* studies have detected the presence and/or quantified the mRNA transcripts produced. This has revealed previously unknown information about the re-initiation [1] and termination steps [2], enhancement of transcription through molecular motors [3,4], and has provided a method to study transcription at a single-molecule level [5].

In high yielding transcription assays, the presence of mRNA can be shown by gel electrophoresis. If the analysis requires quantification of mRNA, then spectroscopy is able to measure the nucleic acid concentration. However, the sample will need to be purified to remove both protein and DNA contamination, which leads to both, a loss of total RNA yield and increased experimental error. Furthermore, both approaches require a high yield of mRNA and therefore, are not typically suitable for eukaryotic transcription assays. The latter typically use radioactively tagged nucleotides [6,7] or Reverse Transcription quantitative PCR (RT-qPCR) is a very sensitive method to quantify transcripts [8]. However, these are multistep processes which can lead to an increase in experimental error. More recently, highly sensitive and selective fluorescent assays, such as Qubit, have become available. However, these are coupled to relatively high costs and the need for specific spectrophotometer systems. Therefore, a need for a low cost, sensitive, easy to use reagent which can be added to the sample to directly compare *in vitro* transcription reactions without additional purification steps could be advantageous.

Fluorescence biosensors have been successfully employed in such roles in various biochemical assays [[9], [10], [11], [12]]. A fluorescently labelled SSB protein from *Escherichia coli* has been successfully used as a ssDNA biosensor for monitoring helicase activity [[13], [14], [15], [16], [17]]. *E. coli* SSB is a well characterised homo-tetrameric protein containing 4 OB-fold domains [18]. It has two main binding modes, known as (SSB)_65_ and (SSB)_35_ [19]. Interestingly, it has been also reported to bind to RNA [[20], [21], [22]].

Here, we aim to expand the application of the SSB biosensor, showing that, along with ssDNA, it can also be used to measure mRNA. This will provide a low cost, rapid alternative for directly measuring mRNA with minimal substrate isolation. To display the functionality and versatility of the biosensor, we use the SSB assay to investigate how the motor activity of myosin VI is required during RNAPII transcription.

## 2 Materials and methods

### 2.1 Reagents

Unless stated otherwise, all reagents were purchased from Sigma Aldrich. Oligonucleotides are listed in Supplementary Table 1.

### 2.2 Protein expression and purification

SSB(G26C) was expressed from pET151 in *E. coli* BL21 DE3 cells. The cells were grown in LB media supplemented 100 μg mL^−1^ ampicillin. IPTG was added to a final concentration of 1 mM and cells were grown overnight at 18 °C. The cells were harvested by centrifugation and re-suspended in 50 mM Tris⋅HCl (pH 7.5), 200 mM NaCl, 1 mM DTT, 20% Sucrose and 40 mM imidazole, supplemented with 1 mM PMSF.

For purification, the cells were lyzed by sonication and protein was purified from the soluble fraction by affinity chromatography (HisTrap FF, GE Healthcare). The pooled protein was further purified through a Superdex 200 16/600 column (GE Healthcare) equilibrated with 50 mM Tris⋅HCl (pH 7.5), 1 mM DTT and 150 mM NaCl. **The purest fractions were concentrated by centrifugation and stored at -80 °C.**

### 2.3 Labelling with MDCC

Labelling was adapted from Ref. [14]. 3 mg of SSB was incubated with 1M DTT for 20 min at room temperature. DTT was removed using a PD10 desalting column (GE Healthcare), equilibrated in labelling buffer (20 mM Tris.HCl pH 7.5, 1 mM EDTA, 500 mM NaCl and 20% glycerol). A 2-fold molar excess of MDCC (*N*-[2-(1-maleimidyl)ethyl]-7-diethylaminocoumarin-3-carboxamide) was added and incubated for 4 h at room temperature, with end-over-end mixing, while protected from light. Excess dye was removed using a PD10 desalting column equilibrated in labelling buffer.

The concentration of SSB was taken using absorbance at 280 nm (A_280_), with extinction coefficient of *ε* = 28,500 cm^−1^ M^−1^ per monomer. MDCC concentration was determined using absorbance at 430 nm (A_430_), with extinction coefficient of *ε* = 44,800 cm^−1^ M^−1^. Labelling efficiency was calculated using equation (1).

(1) $$Labelling\;efficiency=\;\frac{A_{x}}{\varepsilon }\;\times \;\frac{MW\;of\;protein}{mg\;protein/mL}=\;\frac{moles\;of\;dye}{moles\;of\;protein}$$

where *A*_x_ is the absorbance value of the dye at the absorption maximum wavelength and *ε* is the molar extinction coefficient of the dye at absorption maximum wavelength.

### 2.4 Electrophoretic mobility shift assay (EMSA)

50 nM SSB was incubated with 250 nM ssDNA_70_ or ssRNA_70_ for 20 min at room temperature in 50 mM Tris.HCl pH 7.5, 100 mM NaCl, 3 mM MgCl_2_. Samples were loaded onto an acrylamide gel (12% acrylamide, Tris. Boric acid pH 7.5, 2.5 mM Mg) (TBM) and ran in TBM buffer. SYBR^®^Gold (Invitrogen) stained the nucleic acids following the manufacturer's instructions.

### 2.5 Tryptophan fluorescence titration

ssDNA_70_, or ssRNA_70_, were titrated into 200 nM SSB at 25 °C in 50 mM Tris.HCl pH 7.5, 200 mM NaCl and 3 mM MgCl_2_. Tryptophan fluorescence was measured using a Cary Eclipse Fluorescence Spectrophotometer (Agilent), with excitation at 285 nm and emission at 325 nm. To calculate the fluorescence quenched (%) we used equation (2).

(2) $$Fluorescence\;quenched\;\left(\%\right)=\;\frac{F_{i}\;\times DF_{i}}{F_{0}}\times 100$$

where *F*_*0*_ is initial fluorescence intensity, *F*_*i*_ is the intensity after titration and *DF*_*I*_ is the dilution factor from the titration. The titration curves were fitted to equation (3):

(3) $$\left[Quenched\;\%\right]=\;\frac{Amplitude\cdot \left[Nucleic\;Acid\right]}{K_{d}+\;\left[Nucleic\;Acid\right]}+Background$$

### 2.6 Titrations of oligonucleotides to MDCC-SSB

All reactions were performed at 25 °C in a buffer containing 50 mM Tris.HCl pH 7.5, 3 mM MgCl_2_ and 100 mM, or 200 mM NaCl, with 50 nM MDCC-SSB in a final volume of 100 μL. Measurements were performed using a ClarioStar Plate Reader (BMG Labtech). Fluorescence excitation was measured from 400 to 440 nm, with a step-width of 1 nm and emission at 470 nm. Fluorescence emission was measured from 455 to 550 nm, with a step width of 1 nm and a fixed excitation of 430 nm.The fluorescence intensity was then taken at 471 nm. Fluorescence change is presented as a ratio using equation (4).

(4) $$Fluorescence\;change=\frac{F_{i}\times DF_{i}\;}{F_{0}}\;\;$$

where *F*_*0*_ is initial fluorescence intensity at 471 nm and *F*_*i*_ is the intensity at 471 nm after titration. *DF*_*i*_ is the dilution factor for that titration. The curves were fitted to equation (4).

### 2.7 In vitro transcription and RT-qPCR

T7 *in vitro* transcription was performed using the HiScribe™ T7 RNA Synthesis Kit (New England Biolabs) with pET28-RecD2 as a template following manufacturer's instructions. RNAPII *in vitro* transcription was performed using the HeLaScribe kit (Promega). The DNA template was the pEGFP-C3 linearized plasmid containing the CMV promoter which would generate a 130-base run-off transcript. Reactions were performed according to the manufacturer's instructions. The reactions were performed for 60 min at 25 °C. Reactions were also performed following pre-clearance of myosin VI from the sample using an anti-myosin VI antibody (Sigma HPA035483-100UL), RNAPII (Abcam Ab5131) or Dab2 (Abcam ab33441). Protein G Dynabeads (Invitrogen) were prepared according to manufacturer's instructions before being loaded with 4 μg antibody. Samples were incubated for 30 min on ice and beads were extracted immediately before performing the transcription reaction. Where required, 25 μM of the myosin VI inhibitor 2,4,6-Triiodophenol (TIP) was added to the reaction mixture.

RNA was then purified using RNeasy^®^ kit (Qiagen), or Gene Jet RNA purification kit (Thermo scientific), according to manufacturer's protocol. RT-qPCR was performed with one-step QuantiFast SYBR Green qPCR kit (Qiagen) using primers in Supplementary Table 2. RT-qPCR samples were calibrated against known concentrations of the template.

### 2.8 Cell culture and gene expression analysis

MCF-7 cells were cultured at 37 °C and 5% CO_2_, in Gibco MEM Alpha medium with GlutaMAX (no nucleosides), supplemented with 10% heat-inactivated Fetal Bovine Serum (Gibco), 100 units/ml penicillin and 100 μg/ml streptomycin (Gibco). For myosin VI inhibition experiments, MCF-7 monolayers were seeded to 30–50% confluency and then subjected to 25 μM TIP for 4 h. Cells were then harvested for RT-qPCR analysis, as described above.

## 3 Results

By building upon the work by Dillingham *et al* [14], this study used the same SSB mutant, G26C. This mutation does not affect the DNA binding of SSB, nor the formation of its tetrameric state [14]. This SSBG26C mutant will be referred to as SSB throughout this study.

**Fig. 1:**
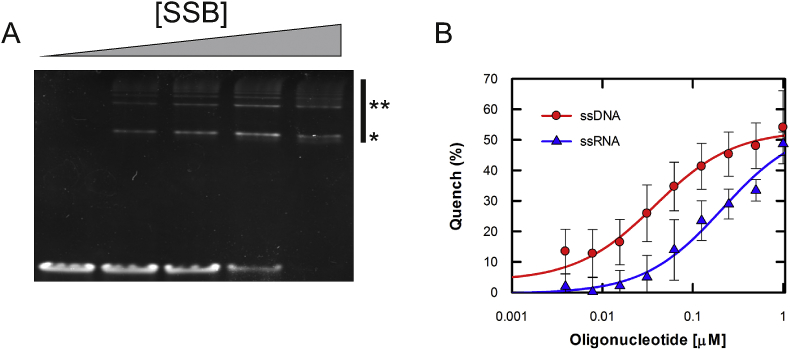
**SSB can bind to single stranded RNA**. (A) Representative EMSA showing qualitative association of SSB with a 70 base ribonucleotide substrate. Bound species are depicted by *. (B) Tryptophan quenching monitored while titrating ssDNA (red circles) or ssRNA (blue triangles). The curves were fitted as described in the methods. Errors bars represent SEM. (For interpretation of the references to colour in this figure legend, the reader is referred to the Web version of this article.)

SSB has been previously reported to bind RNA [[20], [21], [22]]. However, the protein was reported not to bind a polyU RNA substrate [14]. Therefore, we first assessed whether SSB can bind to mRNA. To this end, we initially performed qualitative electrophoretic mobility shift assays (EMSA) with ssDNA_70_ and ssRNA_70_ (See Supplementary Table 1 for sequence). Indeed, SSB bound to ssRNA_70_ in a manner indistinguishable from that of ssDNA binding (Fig. 1A).

To further confirm that SSB does bind to mRNA, tryptophan fluorescence quenching was used. Tryptophan residue 54 was found to be directly involved in binding to ssDNA [23], resulting in fluorescence quenching. Titration of ssDNA_70_ yielded a 50% quenching, with the apparent equilibrium dissociation constant (*K*_d_) being limited by the concentration of SSB in the reaction (Fig. 1B). This was depicted by a breakpoint in the linear phase once the stoichiometric complex of 1 tetramer to ssDNA was reached. This was expected for the concentration of SSB and DNA used here, which are well in excess of the low nanomolar *K*_d_ for the SSB-ssDNA interaction [24]. The ssRNA_70_ yielded a similar quenching response, albeit the apparent affinity was weaker (*K*_d_ ∼200 nM) (Fig. 1B). The weaker binding is agreement with previous findings [20], whereby SSB has preference for ssDNA over ssRNA, although binding is still possible. The consistent quenching response is indicative of a single binding site for both ssDNA and ssRNA, as would be expected from the inspection of the structure. Overall, we can conclude that RNA is a viable substrate for SSB and therefore, it is a suitable for acting as an mRNA biosensor.

**Fig. 2:**
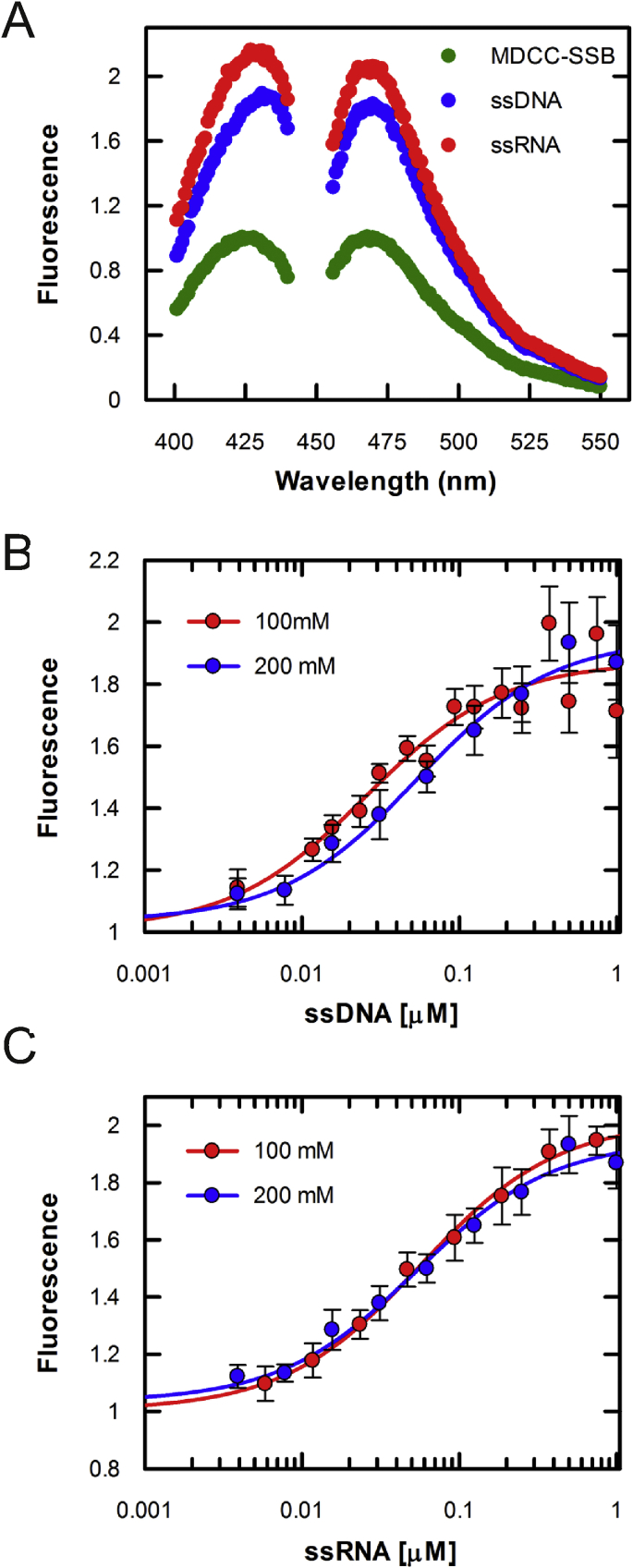
**MDCC-SSB is an mRNA biosensor**. (A) Fluorescence excitation and emission spectra for MDCC-SSB measured in the apo (Green), ssDNA bound (blue) and ssRNA bound (red) states. (B) MDCC-SSB fluorescence monitored while ssDNA was titrated into the biosensor in 100 mM NaCl (red) and 200 mM NaCl (blue). (C) Titration performed as in B but with ssRNA. Errors bars represent SEM. (For interpretation of the references to colour in this figure legend, the reader is referred to the Web version of this article.)

To generate the fluorescent biosensor, we first needed to select a fluorophore. We opted to use the commercially available and environmentally sensitive fluorophore, MDCC. This fluorophore has similar properties to *N*-[2-(iodoacetamido)ethyl]-7-diethylaminocoumarin-3-carboxamide) (IDCC) which was used for the already published ssDNA biosensor [14]. Here, the MDCC-SSB biosensor responded to the addition of both ssRNA_70_ and ssDNA_70_ substrates, as demonstrated by the fluorescence spectra (Fig. 2A). A 1.9-fold increase was observed when excess ssDNA_70_ was added to MDCC-SSB, whereas a 2.1-fold increase was observed upon addition of ssRNA_70_. As with the quenching experiments, the MDCC-SSB response is identical with ssDNA and ssRNA.

To determine the suitability of MDCC-SSB as a biosensor to quantify mRNA, we first needed to establish whether there is a dependence between the fluorescence intensity and the ssRNA concentration. Here, we used ssDNA as a positive control. As shown in Fig. 2B, the MDCC-SSB fluorescence increase was dependent on the concentration of ssDNA_70_. As shown by the tryptophan titrations, SSB is a tight DNA binding protein. Therefore, any free ssDNA should be bound by the biosensor. This tight binding theoretically means that a fluorescence signal should increase linearly until a stoichiometric complex is formed, at which point the signal will be saturated. Indeed, when titrating ssDNA_70_ into 50 nM MDCC-SSB tetramers, there was a clear linear phase, reaching saturation at 65 nM. This is consistent with a 1:1 complex between tetramer (and ssDNA_70_. There was a mild salt dependence on the binding affinity, which is typical of DNA binding proteins.

A similar behaviour was also observed with ssRNA_70_ (Fig. 2C). The saturation point was at 101 nM MDCC-SSB tetramers, which was significantly higher than the one observed for ssDNA_70_. The apparent weaker binding is consistent with the tryptophan titrations. Overall, the biosensor displays a linear response over two-orders of magnitude to RNA concentrations (5–500 nM), which is independent from the ionic strength at concentration between 100 and 200 mM NaCl. Such a range is suitable for *in vitro* transcription experiments.

We have demonstrated that the biosensor has the ability to bind to RNA consisting of 70 bases and to respond in a concentration dependent manner. To test whether the biosensor is able to also detect longer lengths of mRNA at various concentrations, we used *in vitro* transcription to generate mRNA transcripts.

**Fig. 3:**
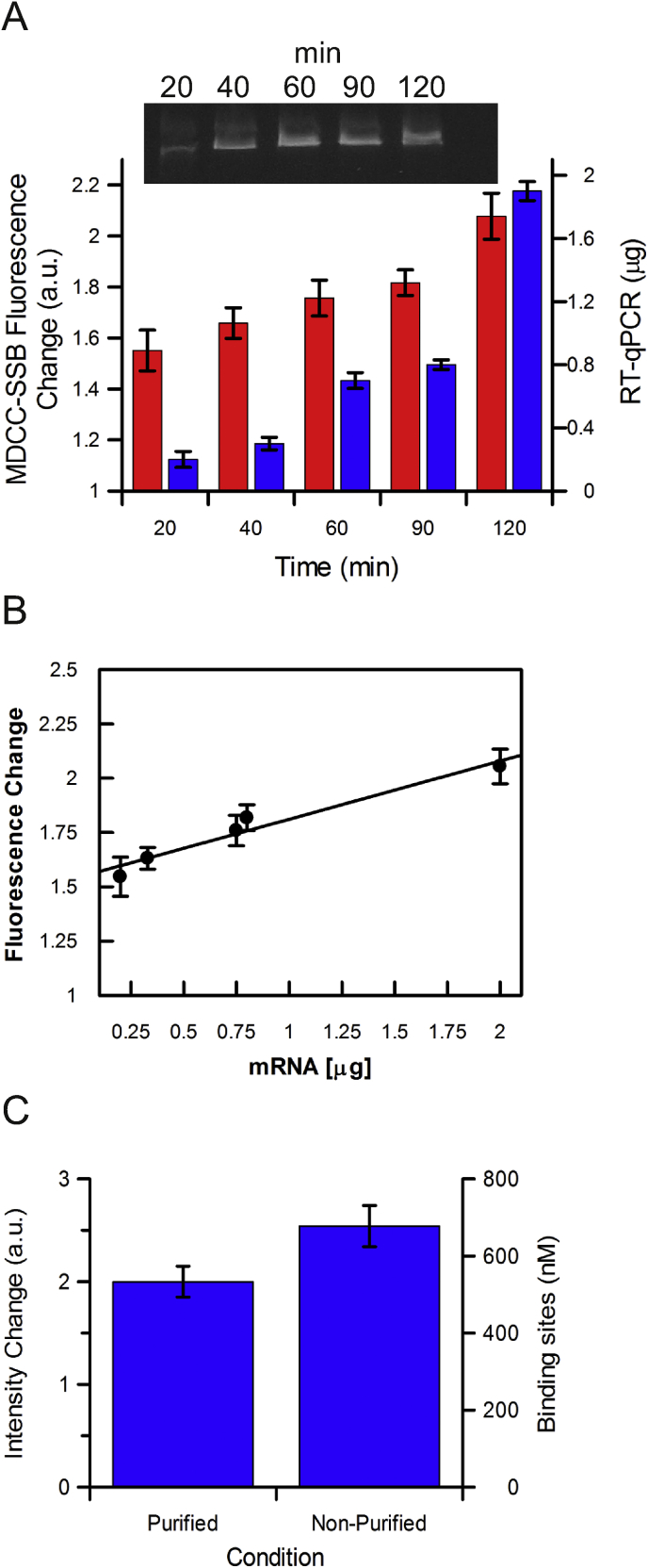
**Application of MDCC-SSB to measure *in vitro* transcription**. (A) Fluorescence enhancement of 1 μM MDCC-SSB with the products from 5 *in vitro* transcription assays. Experiments were terminated at 20, 40, 60, 90 and 120 min before purification. Inset: Gel analysis showing transcript products. Samples were then divided for quantified by RT-qPCR using primers 1 and 2 (Table S2) and analysis by MDCC-SSB. (B) Relationship between MDCC-SSB fluorescence change and mRNA amount determined by RT-qPCR. There is a linear response from the biosensor. (C) MDCC-SSB can bind to both purified mRNA and mRNA present within a transcription reaction. Data is plotted in terms of fluorescence enhancement and concentration of SSB binding sites based upon a calibration in Supplementary Fig. 1. Errors bars represent SEM.

Transcription by T7 polymerase was driven from a T7 promoter which resulted in a 2225 nucleotide run-off transcript. Transcription assays were performed for various amounts of time between 20 and 120 min, to yield different amounts of mRNA products. The products were then purified and quantified by RT-qPCR giving amounts between 0.3 μg and 1.9 μg, yields were confirmed by gel analysis (Fig. 3A). Then, 1 μM of MDCC-SSB was added to each sample of purified mRNA and the fluorescence enhancement was recorded. Fig. 3A clearly demonstrates that the biosensor was able to bind to the generated transcripts. Moreover, there was a linear response in fluorescence intensity versus amount of mRNA, indicating that the biosensor can distinguish differences in transcript yield (Fig. 3B).

To test whether the biosensor can be added directly into the transcription samples, without previous purification of the mRNA products, and to assess the reproducibility of the detection, we setup three *in vitro* transcription reactions to run to completion. At the end of the reaction, the sample was divided in two, half of which was purified and then quantified with RT-qPCR to ensure there was transcription product, whereas MDCC-SSB was directly added to the other half of the sample. Fig. 3C shows that the fluorescence enhancement is 25% higher in the non-purified sample indicating that the biosensor can bind under these conditions and that there is likely to be loss of product during RNA isolation. The fluorescence intensity was also converted into concentration of SSB binding sites of 70 bases (Fig. 3B), using a calibration of MDCC-SSB against known concentration of ssRNA_70_ (Supplementary Fig. 1).

In summary, the SSB biosensor can be used to qualitatively determine differences between *in vitro* transcription assays without the need to purify mRNA. Quantitative analysis is possible through calibrating the fluorescence signal thereby giving concentration of SSB binding sites.

To provide an example application of the biosensor, we used it as a tool to investigate the impact of myosin VI inhibition upon RNAPII transcription. Myosin VI is critical for transcription and myosin VI motor activity is required [3,4].

**Fig. 4:**
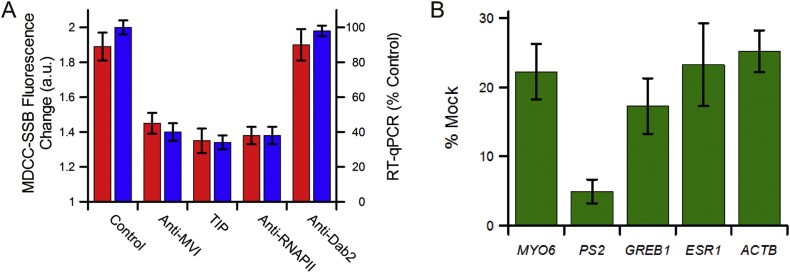
**MDCC-SSB applied to study the impact of myosin VI motor activity upon RNA Polymerase II transcription**. (A) *In vitro* transcription by HelaScribe extracts. Reactions were performed under standard conditions and following antibody depletion, or in the presence of 25 μM TIP myosin VI inhibitor, as described in the Materials and Methods. MDCC-SSB was added to the samples and the fluorescence enhancement was determined under the different conditions. For RT-qPCR, samples were normalized to a non-depleted control reaction (error bars represent SEM). (B) Changes in gene expression following the addition of TIP to MCF-7 cells. Expression is plotted as a percentage of expression in mock cells (Error bars represent SEM).

We revisited the *in vitro* transcription assays performed in Fili *et al* work using HeLaScribe extracts. A 130-base run-off transcript was produced under the control of a CMV promoter. MDCC-SSB was added once the reactions were complete and samples were taken for RT-qPCR analysis. Antibody-depletion of myosin VI has been used to perturb the activity of RNAPII [3,4]. Our results are consistent with these findings, whereby a 60% decrease in MDCC-SSB fluorescence was observed within the myosin depleted sample (Fig. 4A), similar to depletion of RNAPII. The transcription inhibition is specific to protein depletion and not the addition of antibodies. The same observations and conclusions are drawn from the RT-qPCR data. To further explore whether the observed perturbation was specific to myosin VI, we performed measurements in the presence of the small molecule inhibitor, TIP [25]. This inhibitor has been shown to act specifically against myosin VI. Consistent with the depletion, we observed a 70% decrease in transcription, in line with previous results [4]. The inhibition also occurs *in vivo*. We cultured MCF-7 cells in the presence of the myosin VI inhibitor and then monitored the expression of several genes *PS2*, *GREB1*, *ESR1* and *ACTB*. All four genes showed a significant decrease in expression (Fig. 4B), indicating that myosin VI motor activity is required for their expression.

Overall, the SSB biosensor performed in the same manner as the RT-qPCR measurements therefore it can be used interchangeably to assays transcription yield in a qualitative manner. Therefore, the SSB biosensor is a quick, reliable tool which can report on changes with *in vitro* transcription yield.

## 4 Discussion

This study has reinforced the idea that SSB is able to bind to multiple single stranded nucleic acid substrates. The fluorescence increase of the MDCC-SSB biosensor occurs in a substrate concentration dependent manner for both the ssRNA and ssDNA. However, the concentration at which saturation is reached is different between the two substrates. This may imply that there are differences in stoichiometry between RNA and DNA binding. For instance, the 2:1 stoichiometry of RNA:SSB complex could correspond to the 35-base binding mode. It may also indicate that there is a weaker binding affinity for ssRNA. While this would be consistent with the preferential binding to ssDNA, it remains unclear as to whether affinity or binding mode are the cause of this difference. Nevertheless, the qualitative response of MDCC-SSB to ssDNA and ssRNA is similar therefore, the biosensor is suitable for mRNA quantification over two orders of magnitude.

The MDCC-SSB biosensor could be used in two ways: either as a qualitative comparative assay post *in vitro* transcription or as a quantitative assay following calibration. The former relies on the ability of the sensor to generate relative intensity changes between different mRNA amounts. In this case, samples can be normalized to a control and experiments can be matched accordingly. Conversely, the total mRNA concentration can be determined more accurately in terms of SSB binding sites of 65 bases.

Using the MDCC-SSB biosensor offers several advantages compared to the commonly used approach of RNA purification and RT-qPCR, gels or UV-spectroscopy following *in vitro* transcription. These methods are time consuming and lead to a reduced transcription yield, as well as large experimental errors. Conversely, the SSB biosensor can be directly be added at the end of the reaction and readily report on the transcription yield. Commercial fluorescent dyes can be used in a similar manner to SSB but this is achieved with a high cost and the need to dedicated spectrometers.

The benefit of the SSB approach was exemplified by investigating the dependence of transcription on the myosin VI motor activity. The biosensor performance was identical to the RT-qPCR measurements. Consistent with previous findings [4], we found that a small molecule inhibitor of myosin VI successfully decrease transcription to a similar level to when myosin VI is depleted from the reaction. The significance of these findings in a cellular context was demonstrated by exposing mammalian cells to the inhibitor, which then led to a decreased expression of several genes. Such a response is more dramatic than a myosin VI knockdown which did not affect *ESR1* or *ACTB* [4], moreover *PS2* and *GREB1* are reduced to a greater extent. We propose there is a level of redundancy whereby upon knockdown of myosin VI, there is a rescue to maintain transcription levels by another protein, potentially myosin IC [26]. However, the inhibitor stops myosin VI activity and blocks the rescue by preventing additional proteins from binding.

In summary, this study shows that MDCC-SSB is a biosensor suitable for measuring mRNA following *in vitro* transcription. This tool eliminates the need for incorporation of radioactively labelled nucleotides and gel electrophoresis and increases the efficiency of the measurements through its direct use without the need of purification and RT-qPCR. In addition, it can be used to compare conditions without the need for quantification, thereby significantly speeding up the process. Furthermore, its ability to bind RNA implies that the MDCC-SSB can be used to study other biological systems including RNA helicases and potentially other RNA processing enzymes, which makes of this biosensor a useful addition in the currently available toolbox.

## Funding

This work was supported by Medical Research Council [MR/M020606/1] and Leverhulme Trust [ECF-2014-688].
