# Supplementary material for: Application of the SSB biosensor to study in vitro transcription
Source: Biochem Biophys Res Commun. 2018 Feb 12;496(3):820–5. doi: 10.1016/j.bbrc.2018.01.147 (PMC5811048; doi:10.1016/j.bbrc.2018.01.147)
Supplement: Supporting information [file mmc2.docx]

**Application of the SSB biosensor to study *in vitro* transcription**

**Alexander Cook^1^, Yukti Hari-Gupta and Christopher P. Toseland^1^***

## **Supplementary Table 1: DNA and RNA primers**

| **Name** | **Sequence** | **Modification** | **Supplier** |
| --- | --- | --- | --- |
| dT70 (ssDNA_70_) | TTTTTTTTTTTTTTTTTTTTTTTTTTTTTTTTTTTTTTTTTTTTTTTTTTTTTTTTTTTTTTTTTTTTTT | none | Sigma Aldrich, Dorset, UK |
| ssRNA_70_ | UUAGUUGUUCGUAGUGCUCGUCUGGCUCUGGAUUACCCGCUUAGUUGUUCGUAGUGCUCGUCUGGCUCUG | none | Eurofins, Kent, UK |

**Supplementary Table 2: RT-qPCR Primers**

| **Number** | **Sequence** | **Name** |
| --- | --- | --- |
| 1 | GGGTCAACAAAGTCCGTTTC | T7 RT-qPCR For |
| 2 | GTTGACCACCCGGTACTGGTA | T7 RT-qPCR Rev |
| 3 | CATGGAGAACAAGGTGATCTG | TFF1/PS2 qPCR For |
| 4 | CACTGTACACGTCTCTGTCTG | TFF1/PS2 qPCR Rev |
| 5 | ATGGGAAATTCTTACGCTGGAC | GREB1  qPCR For |
| 6 | CACTCGGCTACCACCTTCT | GREB1  qPCR Rev |
| 7 | AGAGCTACGAGCTGCCTGAC | Human B-Actin qPCR For |
| 8 | AGCACTGTGTTGGCGTACAG | Human B-Actin qPCR Rev |
| 9 | AAGCTTCGATGATGGGCTTA | ESR1 qPCR For |
| 10 | AGGTGGACCTGATCATGGAG | ESR1 qPCR Rev |
| 11 | CCGAGCTCATCAGTGATGAGGC | Myosin VI qPCR For |
| 12 | CCAAGCATGATACACTTTTAGTCTCC | Myosin VI qPCR Rev |
| 13 | AAGGGCATCGACTTCAAGGA | RNAPII RT-qPCR For |
| 14 | GGCGGATCTTGAAGTTCACC | RNAPII RT-qPCR Rev |


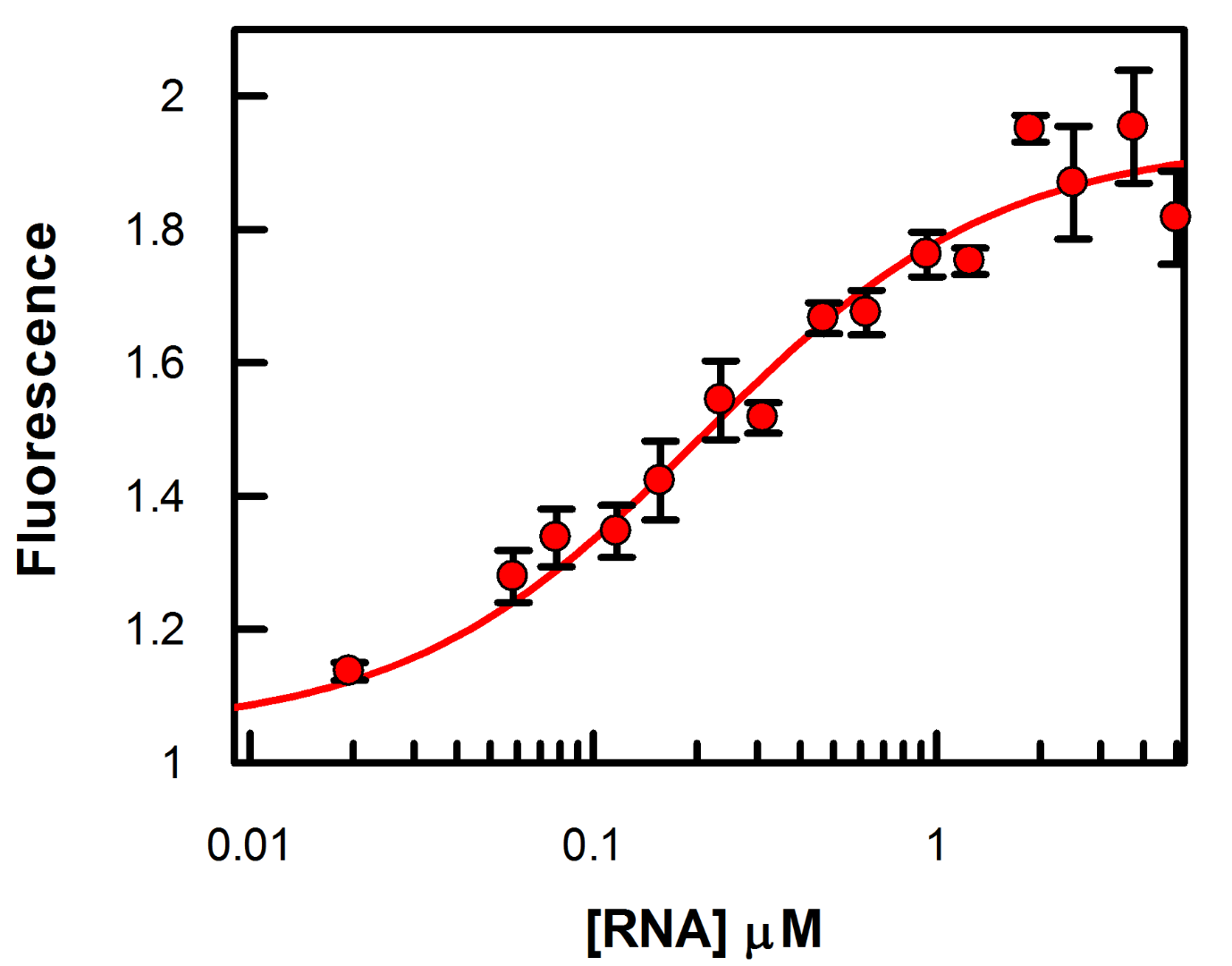


**Supplementary Figure 1: MDCC-SSB Calibration for *in vitro* Transcription.** MDCC-SSB fluorescence monitored while ssRNA was titrated into 1 μM biosensor in 100 mM NaCl. Errors bars represent SEM.
